# Multivariate Analysis of Concrete Image Using Thermography and Edge Detection

**DOI:** 10.3390/s21217396

**Published:** 2021-11-07

**Authors:** Bubryur Kim, Se-Woon Choi, Gang Hu, Dong-Eun Lee, Ronnie O. Serfa Juan

**Affiliations:** 1 Department of Robot and Smart System Engineering, Kyungpook National University, 80, Daehak-ro, Buk-gu, Daegu 41566, Korea; brkim@knu.ac.kr; 2 Department of Architectural Engineering, Daegu Catholic University, Hayang-ro 13-13, Hayang-eup, Gyeongasan-si 38430, Korea; watercloud@cu.ac.kr; 3 School of Civil and Environmental Engineering, Harbin Institute of Technology, Shenzhen 518055, China; hugang@hit.edu.cn; 4 School of Architecture, Civil, Environment and Energy Engineering, Kyungpook National University, 80, Daehak-ro, Buk-gu, Daegu 41566, Korea

**Keywords:** crack analysis, concrete, cumulative distribution function, edge detection, Sobel edge detection

## Abstract

With the growing demand for structural health monitoring system applications, data imaging is an ideal method for performing regular routine maintenance inspections. Image analysis can provide invaluable information about the health conditions of a structure’s existing infrastructure by recording and analyzing exterior damages. Therefore, it is desirable to have an automated approach that reports defects on images reliably and robustly. This paper presents a multivariate analysis approach for images, specifically for assessing substantial damage (such as cracks). The image analysis provides graph representations that are related to the image, such as the histogram. In addition, image-processing techniques such as grayscale are also implemented, which enhance the object’s information present in the image. In addition, this study uses image segmentation and a neural network, for transforming an image to analyze it more easily and as a classifier, respectively. Initially, each concrete structure image is preprocessed to highlight the crack. A neural network is used to calculate and categorize the visual characteristics of each region, and it shows an accuracy for classification of 98%. Experimental results show that thermal image extraction yields better histogram and cumulative distribution function features. The system can promote the development of various thermal image applications, such as nonphysical visual recognition and fault detection analysis.

## 1. Introduction

The issue of concrete infrastructure deterioration has become a global concern; maintenance or rehabilitation for infrastructure stability is necessary. In addition, unexpected expansion, external forces such as wind [1], and incremental loads contribute to structural aging, which increases maintenance, repair, or replacement costs [2,3,4]. Further, numerous variables can cause concrete infrastructure deterioration, such as mechanical stress, fatigue [5], and chemical and environmental conditions [6]. In the past, assessing infrastructure’s condition was done using human subjectiveness. However, when human subjectiveness is used to perform crack analysis, detection, and evaluation as a manual approach, the frequent outcome is time-consuming and error-prone, especially in large datasets. Psychophysical measurements are used to assess image quality in the perception of visual information by a human observer, which constrains the performance of human vision.

Unmanned techniques have been implemented to improve the accuracy and efficiency of inspection. Digital image processing is a widely used and standard method for detecting and evaluating potential cracks in structures [7,8,9]. An algorithm for concrete compressive strength detection using image processing and artificial neural networks (ANNs) was presented in Reference [10]. The presented method used five specific parameters, concrete samples, and acquired images. Multitarget multiclass defects in concrete structures found in civil infrastructures were classified using a deep multi-deformation-aware attention learning architecture, comprising a multiscale assembly of attention and fine-grained feature-induced attention modules [11]. An image-processing technique to provide concrete regression analysis has been employed [12]; the technique can be an auxiliary tool for destructive and nondestructive testing methods. Reference [13] used a convolutional neural network (CNN) to recognize the orientation of crack segments in concretes. In [14], an enhancement algorithm using the swarm optimization technique with adaptive cumulative distribution function (CDF) based on histogram equalization (HE) was proposed for iteratively optimized contrast enhancement and HE, which resulted in a combined function that converted the input images into quality images. An imaging-based detection technique for mapping crack damages in concrete structures has also been introduced [15]; this technique employed the gray-level co-occurrence matrix texture analysis approach integrated with ANN as a classifier to obtain surface damage information. Reference [16] presented an automated crack assessment and quantitative growth monitoring employing dual CNNs. An artificial-intelligence-empowered pipeline for image-based inspection of concretes was illustrated in [17]; this innovative approach comprised anomaly detection, extraction, and defect classification of regions from large image datasets. A review and model comparison using a machine learning (ML) approach for crack detection on a selective scale are presented in [18]. Additionally, Reference [19] used image processing to conduct an experimental study on microscopic mobbing characteristics of pedestrians in built corridors to provide the real-time characteristics of the structures with pedestrian interaction. The fundamental purpose of this research is to provide an image analysis that presents the graphical characteristics linked to images, such as histograms, which can be used for further study. Additionally, images in grayscale offer information on identifying the many types of objects that are included in images and good description of the surfaces of the objects that are present. Thermal imaging was also considered, which provides a more significant image than images taken from an ordinary camera. The image analysis algorithms were utilized for classification, defect detection, and segmentation, and neural network was used for pattern recognition. Both were used for image segmentation and pattern recognition with minimal human intervention.

To solve the issues mentioned, we focus on establishing sufficient information on images to analyze concrete conditions. In addition, to achieve the research objective, an image segmentation approach is implemented to enhance acquired images to simplify the study of the concrete conditions, and an ML technique is employed for detecting concrete defects. In the proposed method, the acquired images are initially preprocessed to remove unwanted information. Then, edge detection (ED) and filtering are used to highlight only the defect region on concrete images. The remainder of this paper is structured as follows: Section 2 addresses the related works. The proposed method is discussed and described in Section 3. Section 4 presents simulations, evaluation results, and interpretations. Finally, Section 5 concludes this study.

## 2. Review of Related Literatures

### 2.1. Image-Processing Analysis

Image processing is a technique for improving an image or extracting relevant information from it. Digital image-processing methods enable the alteration of digital images using computers. Fundamental image parameters include resolution, contrasts, dynamic range, and the signal-to-noise ratio. Details of these parameters are provided in Appendix A.

**Figure 1:**
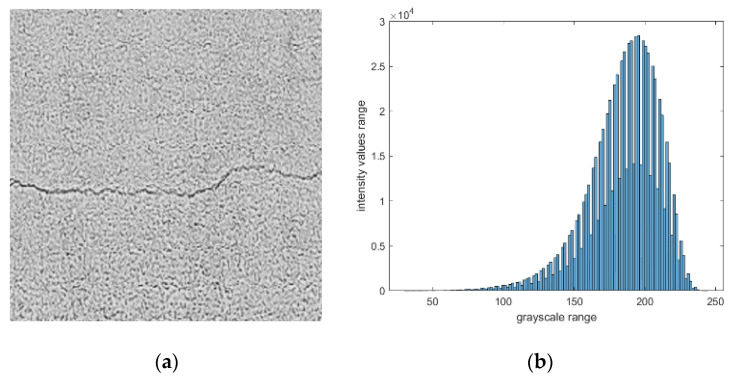
Sample concrete image: (**a**) image in grayscale mode and (**b**) its corresponding histogram.

The representation of an image can take several forms, which can vary in regards to their color space (including aspects such as hue, saturation, or value) and even in graphical schemes. This representation conveys information, such as color, coded information, temperature mapping, and how an image is digitally preserved. Cumulative histograms and the cumulative distribution function (CDF) are the two common graphical representations of digital image processing. The detailed description of these two graphical representations is presented in Appendix A. Figure 1 shows the corresponding histogram of a sample image.

**Figure 2:**
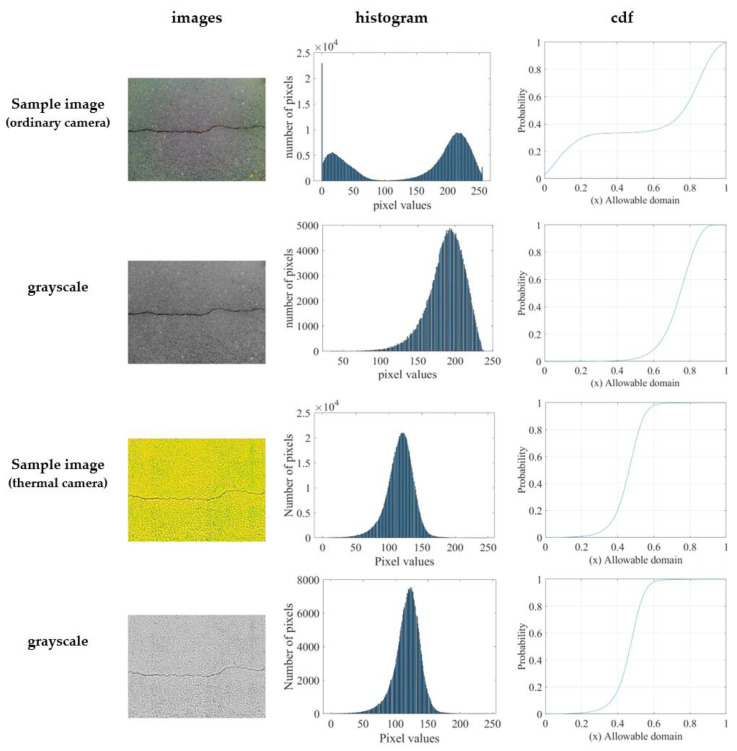
Histogram and CDF comparison on both sample images (taken from an ordinary camera and a thermal camera, respectively).

Figure 2 shows the histogram and CDF comparison between two images taken from an ordinary and a thermal camera, respectively. The image histogram was plotted using pixel values and the number of pixels. The histogram of the sample image taken from the ordinary camera exhibits a bimodal distribution. One peak represents the object pixels, whereas the other peak represents the background. Meanwhile, the histogram of the thermal image shows a good distribution of pixels over the entire intensity range. The histogram also shows most pixel values clustered in a small area; the top half of the intensity values are occupied by only a few pixels. The more the pixels are evenly distributed over the entire intensity range, the more easily the image can be transformed. The CDF is more linear in the thermal image, producing a more enhanced image. Thus, the representation of a thermal image in a different domain from the extracted features such as the histogram and CDF is helpful for postprocessing, e.g., in pattern selection, which can be used for classifying and assessing an image.

**Figure 3:**
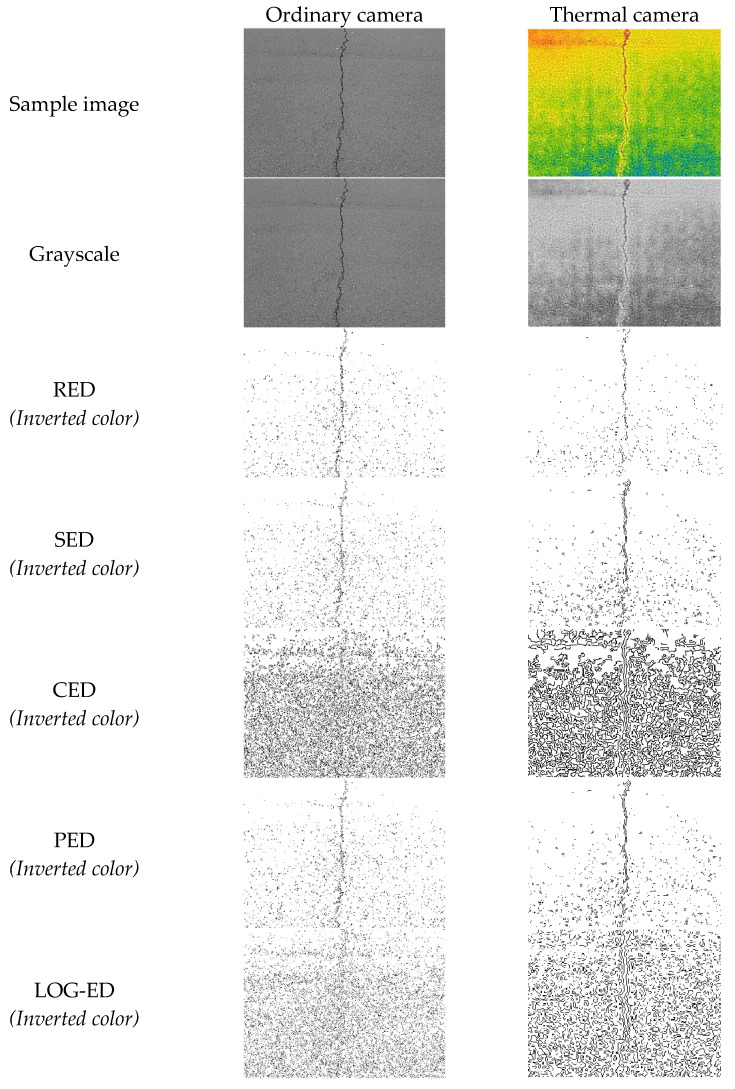
Comparison of sample images taken from ordinary and thermal cameras.

Using an image edge detection (ED) method, the object boundaries can be established on a per-image basis. These methods can be helpful for examining individual pixels and their nearby segments to determine portions of an image that have strong contrast. The method of identifying edges in image processing is known as ED [20]. Image segmentation is a necessary step in image analysis. The segmentation process separates an image into its components or objects that have the same texture or color. In this study, only Sobel edge detection is used in the simulation process. A brief discussion of edge detection is presented in Appendix A. Figure 3 shows the simulated results using the different ED techniques compared between an image dataset taken from an ordinary camera and a thermal camera. It shows that the output from the thermal camera is better than that from the ordinary camera.

### 2.2. Related Implementation

Below are some related works for concrete analysis that use image-processing techniques. Reference [21] described an online image-processing-based technique for rapidly and non-invasively detecting cracks in pressed-panel goods; however, this method used a standard camera, and the dataset was relatively small. An algorithm developed in [22] presented a feature detection approach that uses the Sobel operator to filter and denoise concrete images before implementing the Otsu method for thresholding segmentation for crack edge identification. Another implementation that uses the ED technique was presented in [23]; it analyzes crack identification for bridges. The presented work compared the crack detection results using the fast Fourier transform, Sobel filter, and Canny filter. A comparison of performance using deep CNNs and EDs for image-based crack detection in concrete structures yielded an 86% accuracy rate in the network that correctly detected the cracked images [24]. Finally, Reference [25] presented a CNN application for ground-penetrating radar images that automatically recognized, located, measured, and provided a three-dimensional reconstruction of concealed cracks. In addition, three distinct CNNs were constructed to automate the tasks mentioned above: recognition, location, and feature extraction.

## 3. Methodology

**Figure 4:**
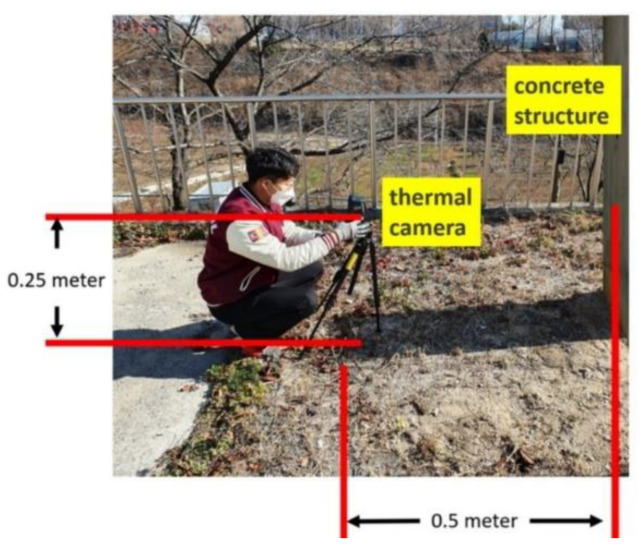
Physical setup for image acquisition.

Below are presented the detailed technical specifications, algorithm, and setup used in this paper. Table 1 under Experimental Setup provides the technical specifications of the thermal camera used in this study. The specifications for the physical setup in data acquisition are shown in Figure 4.

Experimental Setup.

Data Acquisition Setup.

### 3.1. Image Acquisition

The acquired dataset consists of 2700 thermal concrete images from various structures of universities in Daegu City, Republic of Korea, between November 2019 and September 2021. The proposed approach was simulated using the MATLAB platform. Detailed descriptions of the algorithm and experimental setup are discussed below.

### 3.2. Algorithm

**Figure 5:**
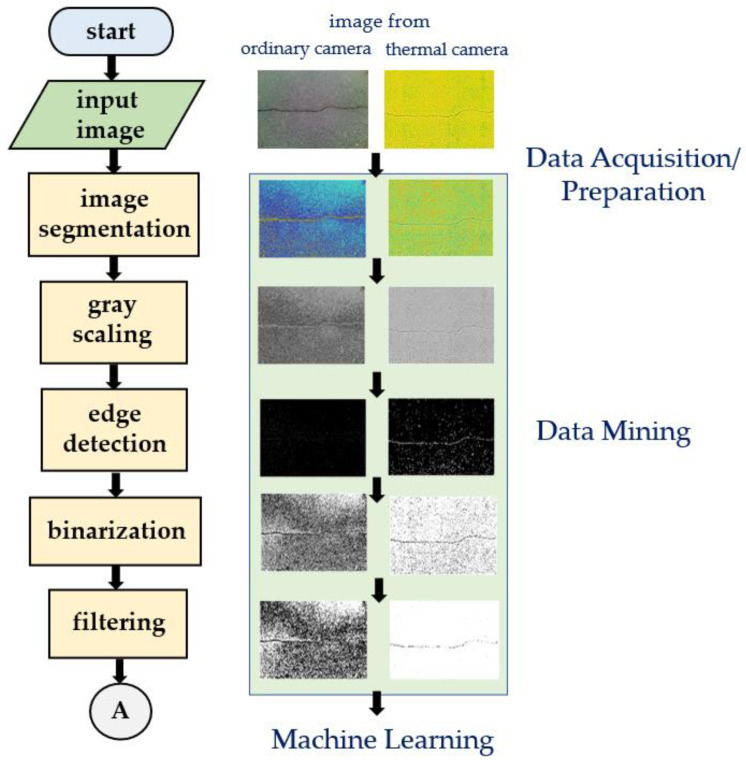
Proposed flowchart of the image-processing techniques.

**Figure 6:**
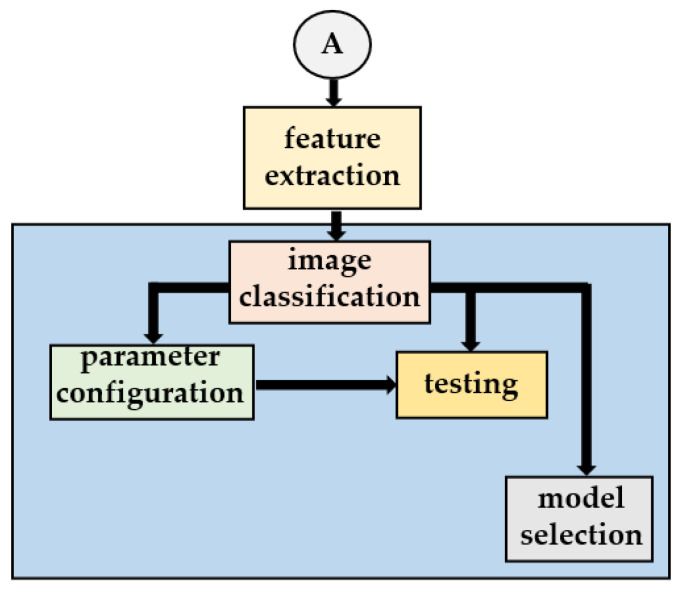
Proposed processing blocks of the neural network.

The following sections describe each step of the proposed work. Pre-image processing was implemented to improve the raw image. All concrete images were then enhanced using different image-processing techniques as shown in Figure 5. Finally, a convolutional neural network approach was applied for automatic image classification to assess the accuracy of classification on the testing image, which is shown in Figure 6.

Below are the details of each processing block and the MATLAB syntaxes used in this study.

Step 1: Loading and Reading of Images.

Initially, the raw thermal concrete images were to be imported from the dataset folder. First, the MATLAB function “*dir*” prepared the listing of the files and folders in the current folder for the task. Then, the “*imread*” function was implemented at this stage.

Step 2: Resizing Images.

To visualize the change in the images, two MATLAB functions were implemented. The “*imresize*” function provided the image in the size necessary for the required task. Then, the “*imshow*” function displayed the image for visual verification. In this study, the base size of all sample images was set to a 720 × 576 pixel value (4:3 aspect ratio).

Step 3: Image Segmentation.

Image segmentation is used to transform an image representation into something more meaningful and easier to analyze. This study applied image segmentation to concrete images; only those with cracks were extracted. Furthermore, the clustering method called k-means clustering was performed for the segmentation process with a k value of 4.

Step 4: Grayscale-Level Image.

The resulting image from step 3 was converted to a grayscale image using the MATLAB function “*rgb2gray*,” which removed the hue and saturation content information while maintaining the luminance.

Step 5: ED Techniques.

This step presented the relative performance of ED techniques as SED. The ED technique was implemented and tested using a sample crack image. The objective was to produce a clean edge map by extracting the principal edge features of the image.

Step 6: Morphologic Noise Reduction

Aiming to detect concrete surface cracks, the Otsu algorithm is processed based on differential images. The Otsu method selects a threshold that reduces the intraclass variance of the black and white pixels that have been thresholded.

**Figure 7:**
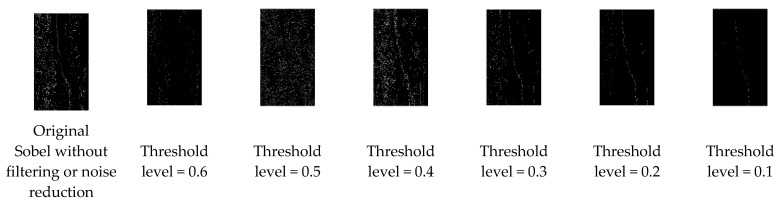
Obtained images using different threshold levels to identify which is appropriate to determine the visible crack region of sample images.

**Figure 8:**
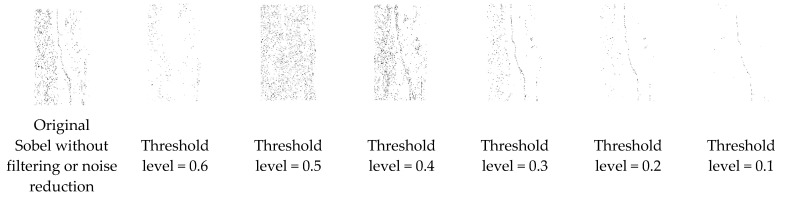
Obtained images were inverted to view the visible crack region of sample images easily.

Figure 7 shows the different threshold iteration levels used in the sample image. The inverted image is depicted in Figure 8.

Step 7: Median Filtering of Grayscale Level.

**Figure 9:**
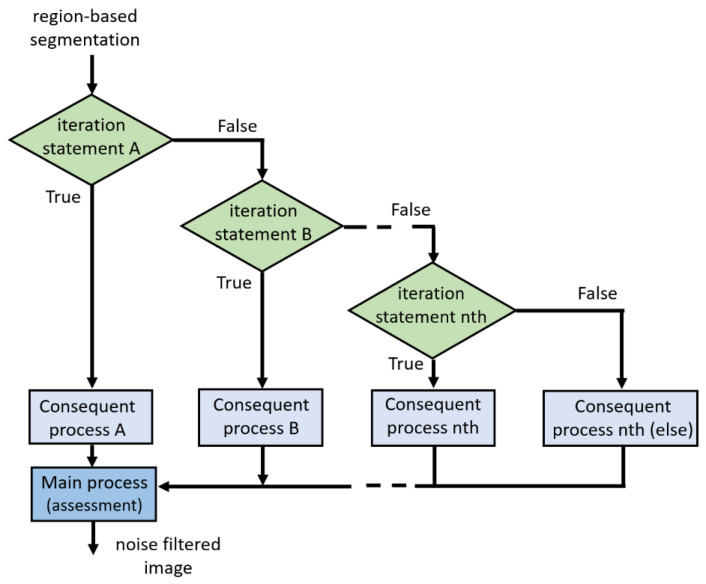
Iteration for noise reduction.

In this study, median filtering was used to minimize noise while preserving the edges of the sample images. The median filter works by moving an image pixel by pixel and changing each value with the median value of the adjacent pixel. The median is calculated by first numerically sorting all pixel values in the window and then replacing the pixel under consideration with the middle (median) value. Further, as shown in Figure 9, a noise reduction algorithm was implemented with an appropriate filter iteration to enhance the sample images being tested, preventing any unnecessary data from being included in the noise reduction process.

**Figure 10:**
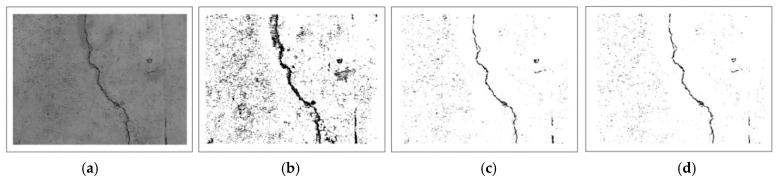
Results of filtering the noise: (**a**–**d**) initial grayscale image to the last transition result.

**Figure 11:**
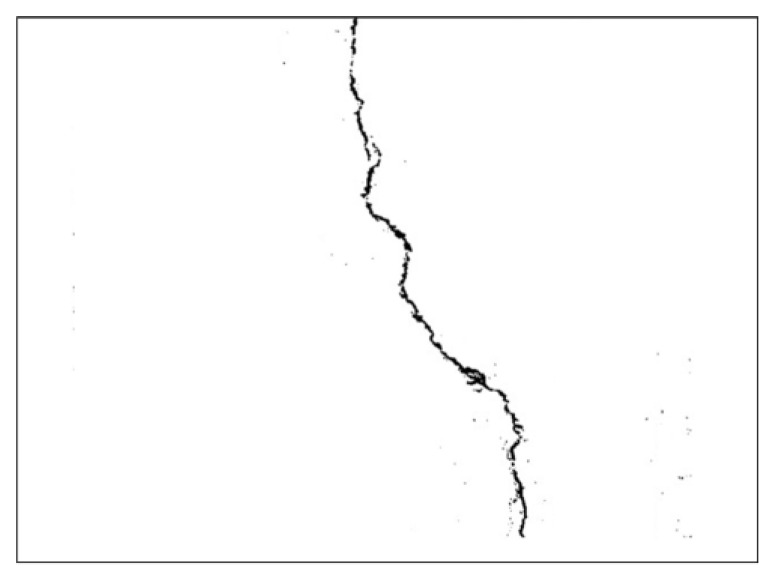
Sample results after the different image processing and noise reduction iteration.

As seen in Figure 10, the original image was further filtered to remove any unwanted noise in the target object of the image. Additionally, a clearer version of the filtered output image is shown in Figure 11.

### 3.3. Image Classifier

Following feature extraction and image enhancement, the proposed approach employs CNN [26,27] as a feature extractor and a support vector machine (SVM) as a classifier to categorize images. The results of this module section can then be used to pinpoint faults. This module section of the classification’s full description is as follows. The employed CNN can be used as a feature extractor and a classifier in various real-world applications [26]. This study used the Keras sequential model, which included convolutional, activation, and max-pooling layers. The first convolutional layer comprises 32 filters with 3 × 3 pixel dimensions. Following filtering, a ratio of 2 was employed to facilitate the max-pooling procedure.

The convolutional layer’s main job is to detect the local connections of features from the prior layer. The feature map output is subsequently transmitted to the activation layer, which is the ReLU. In vision systems, the max-pooling approach is used for two reasons: (1) to reject non-maximal values, which reduces layer calculation time; and (2) to execute down-sampling operations on 2 × 2 subregions to minimize the dimensions of the intermediate feature vectors. Then, the filters are piled together, and fully connected layers are used to compute the class scores. The output of the fully connected layers is used as the input feature vectors for the SVM classifier in the proposed model. At the final stage of the proposed defect detection, an SVM classifier was used instead of CNN’s softmax layer to find a hyperplane that divides the most significant fraction of a labeled dataset into subgroups appropriate for binary classification. The training data comprise pairs of training samples (x1, y1),...(xi, yi), where xi is the observation or input feature for the ith sample, and yi is the associated class label (x1, 0). The discriminant function that transforms an input feature space xi into a class label yi is the SVM classifier. Since a radial basis function was used as the kernel in SVM, cross-validation was performed to obtain the optimal kernel values. Moreover, the experimental setup was simulated via MATLAB environment for both the ground truth/actual and predicted labels; positive and negative values were assigned to defective and nondefective input images, respectively.

A confusion matrix is a representation of the performance of any classification model on a dataset. For reference, the two possible predicated classes are “yes” and “no”. In this paper, predicting non-defective concrete images means “yes”, while “no” would indicate cracked images. The rows of confusion matrix correspond to the predicted class (Output Class), while the columns correspond to the true class (Target Class). The diagonal cells represent correctly classified observations, and the off-diagonal cells are incorrectly classified observations. The matrix shows both the number of observations and the corresponding observed percentage equivalent to the total dataset number in each cell. Additionally, the row at the bottom of the confusion matrix provides the percentage equivalent of all datasets belonging to each class that are correctly and incorrectly classified. On the other hand, the column of the confusion matrix shows all the predicted percentages that belong to each class that are correctly and incorrectly classified. Lastly, the cell at the bottom right of the confusion matrix shows the overall accuracy of classification. Below are the definitions of each type of cell in a confusion matrix.

True Negative (TN): Predicted “no”; means that cracked images are classified correctly as “defective”.

False Positive (FP): Predicted “yes”; means that cracked images are classified inaccurately as “non-defective”.

False Negative (FN): Predicted “no”; means that non-defective concrete images are classified as “defective”.

True Positive (TP): Predicted “yes”; means that non-defective concrete images are classified correctly as “non-defective”.

Below are equations for the confusion matrix interpretation.

(1) $$Accuracy=\frac{\mathrm{TP}+\mathrm{TN}}{\mathrm{TP}+\mathrm{TN}+\mathrm{FP}+\mathrm{FN}}$$

(2) $$Precision=\frac{\mathrm{TP}}{\mathrm{TP}+\mathrm{FP}}$$

(3) $$Sensitivity=\frac{\mathrm{TP}}{\mathrm{TP}+\mathrm{FN}}$$

(4) $$Specificity=\frac{\mathrm{TN}}{\mathrm{TN}+\mathrm{FP}}$$

(5) $$Negative\;Predictive\;Value=\frac{\mathrm{TN}}{\mathrm{TN}+\mathrm{FN}}$$

For this objective, two ML-based classifiers (SVM and ANN) were employed. A challenging aspect of developing ML-based classifiers is determining their parameters. In this study, the classifier parameters, such as the SVM kernel type, number of ANN layers, and number of neuron-nodes per layer, were correctly chosen based on earlier work [28] and subsequent experimental results.

## 4. Results and Discussion

**Figure 12:**
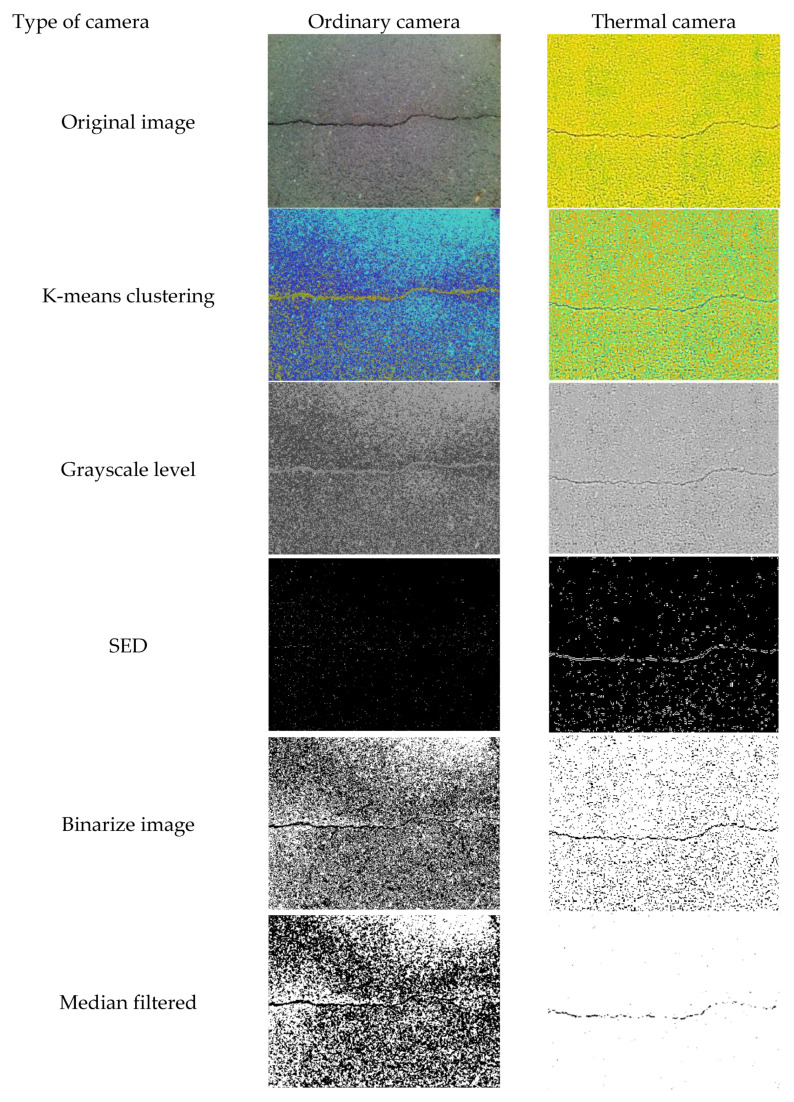
Sample results of the proposed image processing.

Figure 12 illustrated the sample results of each step in the proposed image-processing method, for images taken from both ordinary and thermal cameras. It shows that using a thermal camera for concrete crack image acquisition could provide a better result when the proposed method was implemented. Images taken from both the ordinary camera and the thermal camera were subjected to different digital image processing. The figure illustrates that the thermal-imaging technique of this proposed algorithm is appropriate and can provide additional support for digital image analysis, because the outcome of each stage for the thermal image is much better than that of the image taken from the ordinary camera.

**Figure 13:**
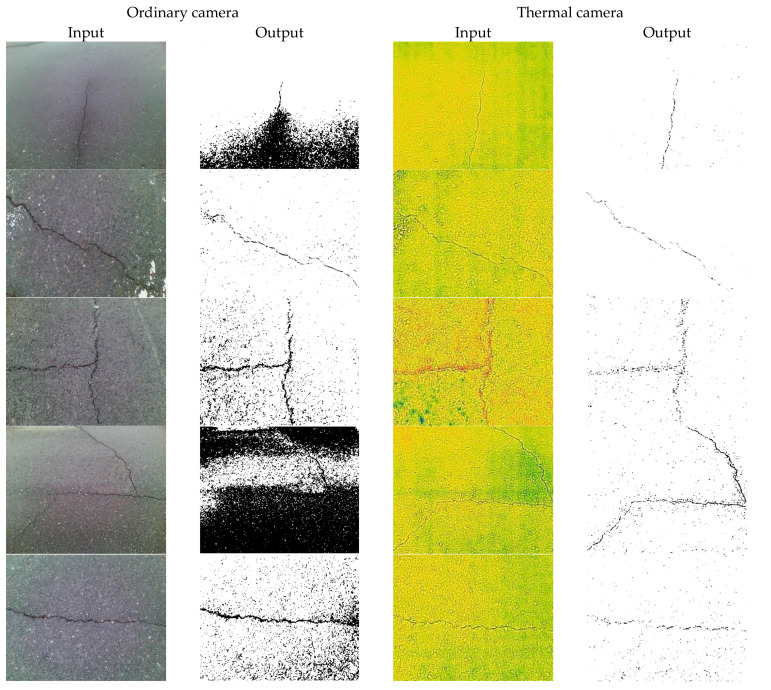
Sample outputs of corresponding inputs from ordinary and thermal cameras.

Figure 13 shows some sample outputs of corresponding inputs, illustrating images taken from both ordinary and thermal cameras. As seen in this figure, by assessing the perceptual quality of the sample input images, various features could be estimated to represent subjective qualities, whose characteristics could be considered desirable or not. Images taken from the thermal camera provided better edge visibility, contrast, and brightness.

### 4.1. Analysis of Five Tonal Zone of Histogram

**Figure 14:**
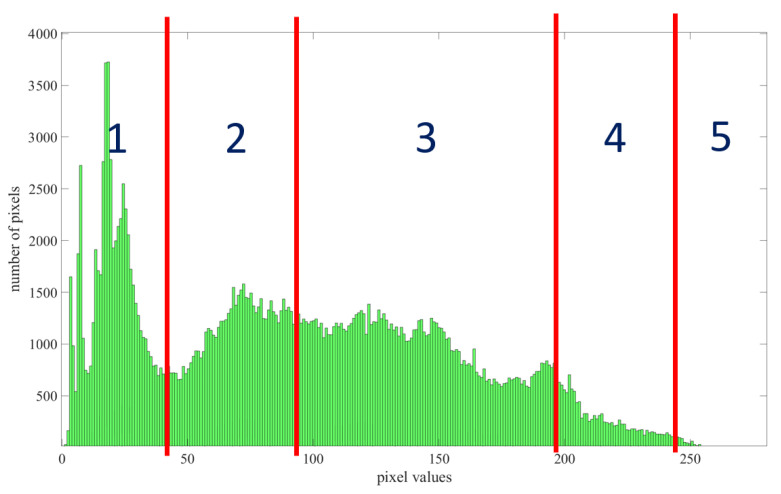
Representation of five tonal zone contents of an image histogram.

Five tonal zones of the histogram are illustrated in Figure 14; these zones are (from 1 to 5) blacks, shadows, midtones, highlights, and whites, corresponding to the tonal range of 0–255.

1. Blacks—this segment is completely black, with no details captured. It also has a very narrow tonal range located on the far left side of the histogram. When a histogram reaches the far left of the chart (tonal range of 0), it means that shadows have been clipped on the image.

2. Shadows—shadows are frequently mistaken for blacks, especially when a darker shadow appears; however, this segment has a slightly wider tonal range than blacks. Shadows have some details and can be lightened to some extent. Usually, image noise appears in this area.

3. Midtones—this segment has the most tonal range and contains the most pixels. Even if there is stretch or shift in any direction, the tones will most likely remain intact.

4. Highlights—this segment has the same property as shadows, but it is in the brighter part of the image and contains some visible details. It can be cautiously adjusted to the far right toward clipping.

5. Whites—whites have a similar characteristic to blacks, but this segment is pure white with no details. If a histogram reaches the far right, it means that more information in the brightest portion has been clipped.

**Figure 15:**
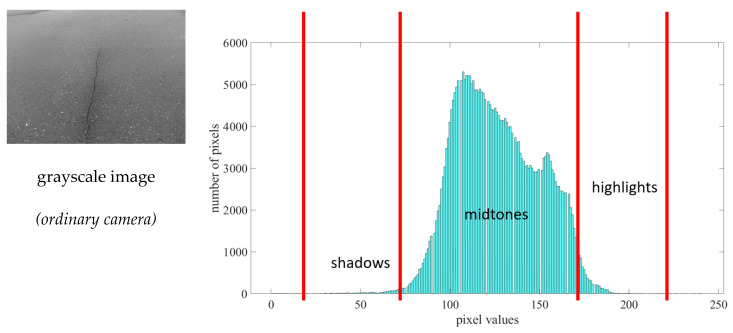
Histogram tonal zone representation of the sample image using an ordinary camera.

Figure 15 shows the tonal zone representation of the image taken from the ordinary camera; a small-scale shadows region appears in the histogram, which indicates that the input image is close to a black segment. In addition, more information is provided in the midtone section, resulting in an asymmetrical histogram. Finally, a small portion of highlights exists in this image, which is likely the same as the shadow contents.

**Figure 16:**
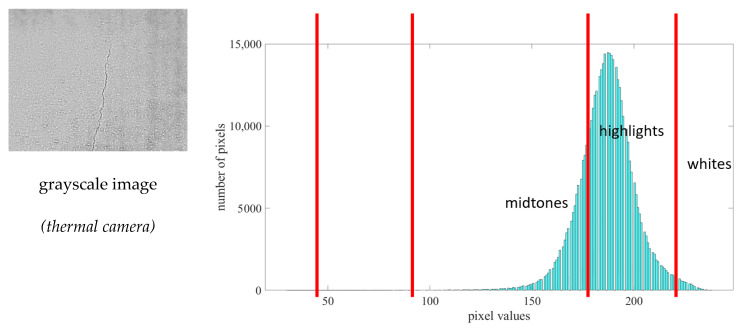
Histogram tonal zone representation of the sample image using a thermal camera.

Figure 16 depicts a symmetrical histogram if shifted further to the far left portion of the image. If shifted to the far left portion, the sample image might have a better contrast quality. In addition, it shows that most pixels fall between the midtone and highlight portions of the image.

### 4.2. Image Quality Metrics

As shown in Table 2, image quality assessment is a difficult task, yet the choice of technique is fundamental for evaluating image quality. Techniques belonging to objective fidelity, such as mean square error (MSE) and peak SNR (PSNR) assessment, and subjective fidelity, which corresponds to the human visual system, such as the multiscale structural similarity (MS-SSIM) index, are widely used. In addition, no-reference algorithms use statistical features of the input image to evaluate image quality, such as blind referenceless image spatial quality evaluators (BRISQUE), natural image quality evaluators (NIQE), and perception-based image quality evaluators (PIQE). The reference image was set using the masking technique for MSE, PSNR, SNR, and MS-SSIM only and was compared with the final output of the proposed image-processing method using the thermal and ordinary cameras.

The experiment results of the CNN and proposed method, which is the CNN–SVM, were compared. Table 3 displays the results of the proposed fault identification scheme for the training dataset. Further, the CNN–SVM method alone outperformed the CNN method for both the training and testing datasets (Table 3 and Table 4).

The correlation value indicates whether the provided dataset is on the best-fit line. In most cases, a positive correlation should be close to +1, and a negative correlation should be close to −1. Moreover, the regression value should be between 0 and 1, with values closer to 1 indicating a model that better fits the dataset. Table 5 shows that the correlation and regression values provided the best model.

## 5. Conclusions

Images of structural sites are frequently used to document construction scenes. The ability to automatically detect material regions in these images can be utilized to automate construction applications, such as monitoring and surface quality assessment. Existing studies necessitated the use of acceptable material classification thresholds. However, they did not elucidate how to determine these levels. This paper presents an algorithm for using thermal imaging technology for concrete image analysis that utilizes different image-processing techniques, with the aim of representing the sample images in an easier-to-interpret domain.

Furthermore, noise reduction techniques were implemented with an appropriate filter iteration to enhance the sample images being tested, preventing any unnecessary data from being included in the noise reduction process. Concrete regions in a sample image can be recognized without the need for manually specifying thresholds. Additionally, in this study, we propose an automated model for detecting concrete regions in images of structural sites. As such, we trained a classifier on 2700 samples.

Moreover, the proposed approach not only uses ordinary images but also uses thermal images. The thermal imaging technology algorithm extracts characteristics from thermal images to simplify their representation into a more manageable area for analysis. In addition to an image histogram, a thermal image provides considerable information during image processing. Experimental results demonstrated that the improved thermal images provided better histogram and CDF features—further, the proposed method employed CNN to improve image classification, with a 98% accuracy. Lastly, the correlation and regression values provided the best model of the proposed concept in the dataset used. The proposed method may encourage the development of various thermal image applications, such as nonphysical visual recognition and fault detection analyses. In the future, many properties of these thermal images could help neural networks in categorization applications.

In the future, we plan to deal with different external factors such as various lightning conditions, high surface roughness, and differences in the concrete surface, and to provide different comparative analyses on how these factors affect the equipment technical specifications and setup. We will also consider sensitivity testing in different models.

## Figures and Tables

**Table 1:** Thermal camera technical specifications.

Brand/Company Name	FLIR E8
Field of View	45° × 34°
Object Temperature Range	−20 °C to 250 °C
Image Frequency	9 Hz
Thermal Sensitivity	<0.06 °C
Accuracy	±2 °C
Thermal Palettes	Iron, Rainbow, Grayscale
File Format	Radiometric JPG
On-board Digital Camera	640 × 480

**Table 2:** Image quality metrics.

	Ordinary Camera	Thermal Camera
images	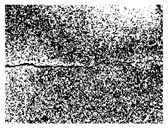	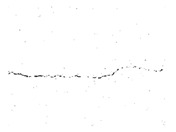
MSE	23,432.0312	240.7319
PSNR	4.4327	24.3155
SNR	4.4053	24.2881
MS-SSIM	0.0321	0.7885
BRISQUE	43.4582	45.0100
NIQE	15.1913	13.2069
PIQE	63.0411	88.7166

**Table 3:** Validation results of the training set based on sensitivity, specificity, and accuracy.

Classifier Method	Percent Sensitivity	Percent Specificity	Percent Accuracy
CNN	98%	94.96	93.96
CNN-SVM	99%	95.3%	98%

**Table 4:** Validation results of the testing set based on sensitivity, specificity, and accuracy.

Classifier Method	Percent Sensitivity	Percent Specificity	Percent Accuracy
CNN	94.25	90%	90%
CNN-SVM	97.55%	93.65%	93.96%

**Table 5:** Correlation and Regression Values of CNN and CNN–SVM.

Coefficient	CNN	CNN–SVM
Correlation	0.9321	0.9981
Regression	0.9629	0.9995

**Figure A1:**
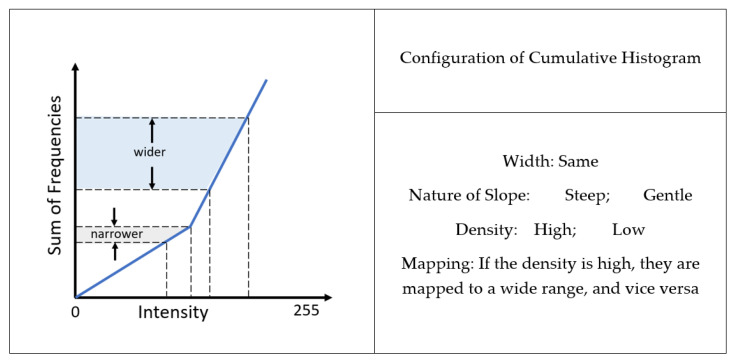
Concept of Cumulative Histogram.

**Figure A2:**
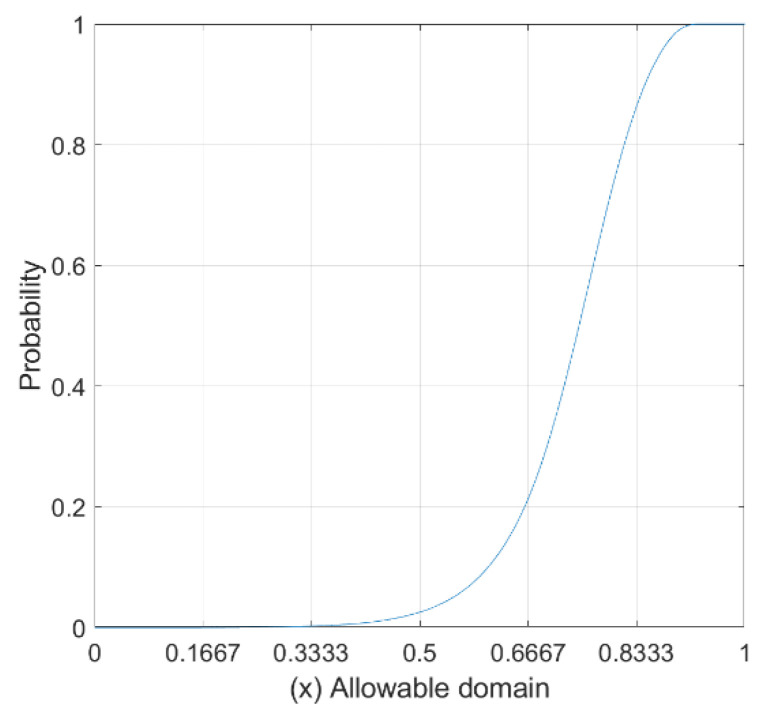
Sample image of a CDF.

**Figure A3:**
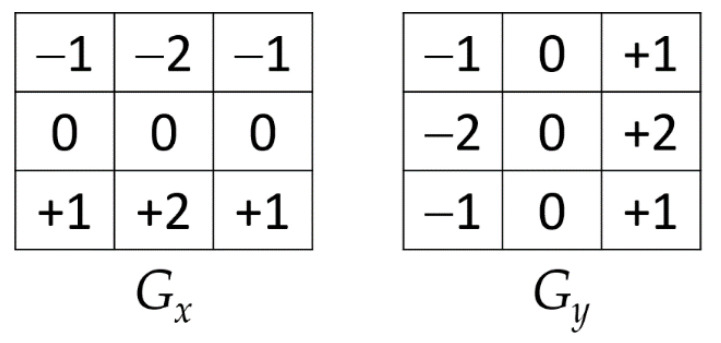
3 × 3 masks of SED.

**Figure A4:**
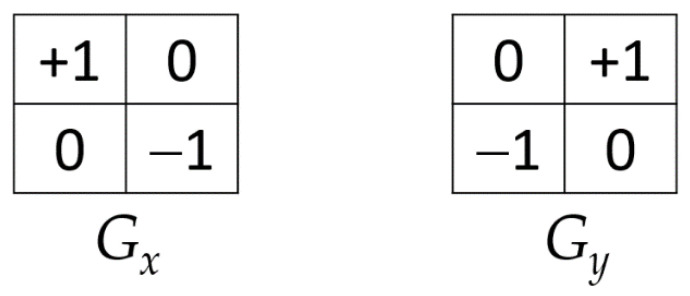
2 × 2 masks of RED.

**Figure A5:**
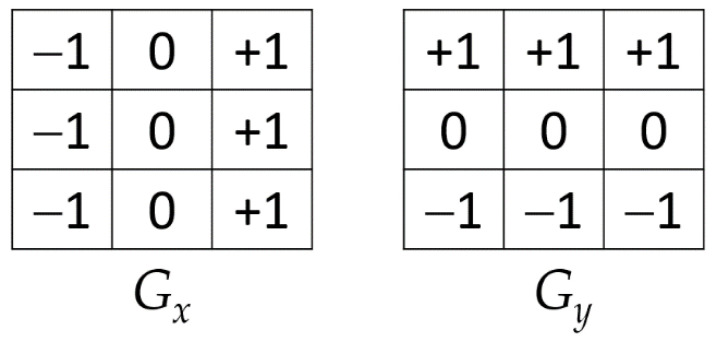
3 × 3 masks of PED.

**Figure A6:**
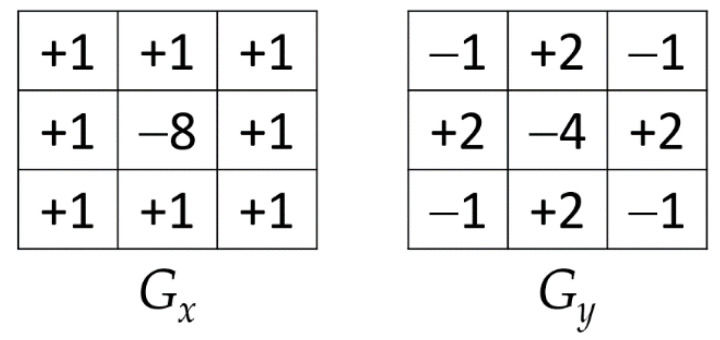
3 × 3 masks of LOG-ED.

**Figure A7:**
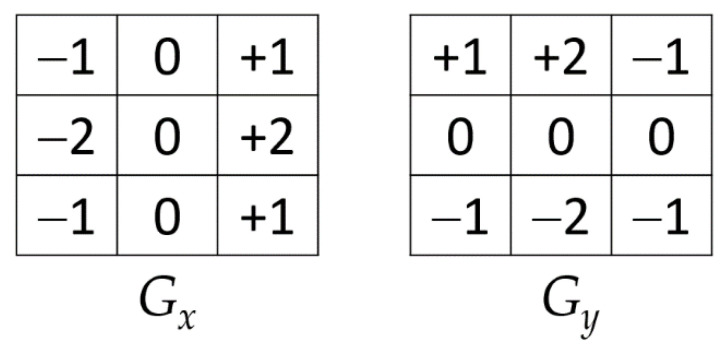
A 3 × 3 masks of a Canny ED technique.

## Data Availability

Data available on request due to restrictions. The data presented in this study are available on request from the corresponding authors. The data are not publicly available due to the project’s contract.

## Abbreviations

CDF	**Cumulative Distribution Function**: this function of image processing presents the resulting image as a linear cumulative distribution function.
CED	**Canny Edge Detection**: this edge detection operator uses a multi-stage algorithm to detect a wide range of edges in images. It locates the intensity gradients of the image and applies non-maximum suppression remove spurious response in the edges.
CNN	**Convolutional Neural Network**: this provides the function of classification of images.
ED	**Edge Detection**: this technique identifies points in a digital image with discontinuities. It sharpens changes in the image brightness.
FN	**False Negative**: this provides the predicted “no”, indicating that non-defective concrete images are classified as “defective”.
FP	**False Positive**: this provides the predicted “yes”, indicating that cracked images are classified inaccurately as “non-defective”.
HE	**Histogram Equalization**: this function is a method in digital image processing that provides contrast adjustment using the histogram of the sampled image.
LOG-ED	**Laplacian of Gaussian Edge Detection**: initially, this smoothens an image, and it then calculates the Laplacian. The process results in a double-edged image. It finds edges and then locates the zero-crossing between the double edges.
ML	**Machine Learning**: this simply predicts outcomes of classifying the sampled images. Machine learning algorithms use historical data as input to predict new output values.
PED	**Prewitt Edge Detection**: this operator is appropriate for detecting the magnitude and orientation of edges. It also has the same parameters as Sobel edge detection; however, it is easier to implement.
RED	**Robert’s Edge Detection**: this operator is a straightforward and efficient approach to quantifying an image’s spatial gradient. The pixel value at a location in the produced image represents the estimated absolute magnitude value of the inputted image’s spatial gradient at that location.
SED	**Sobel Edge Detection**: this operator works by calculating the gradient of the intensity of the digital image at each pixel within the image. It locates the direction of the maximum increase from light to dark and the rate of change in that direction.
SNR	**Signal-to-Noise Ratio**: this function is a general metric for determining image quality. It is described as the relative strength of an aimed signal from a sample compared with the undesired background signal from noise.
TN	**True Negative**: this provides the predicted “no”, indicating that cracked images are classified correctly as “defective”.
TP	**True Positive**: this provides the predicted “yes”, indicating that non-defective concrete images are classified correctly as “non-defective”.

## Appendix A. Fundamental Definitions of Terms Used in This Article

### Appendix A.1. Image-Processing Analysis

Image processing is a technique for improving an image or extracting relevant information from it. Digital image-processing methods enable the alteration of digital images using computers.

#### Appendix A.1.1. Fundamentals of Image Parameters

Below are the core imaging parameters and their definitions:

1. The ability to discern between two (or more) objects close in space is referred to as *resolution*. In digital imaging, resolution refers to the quantity and quality of the pixels in each digital image; the more significant the number of pixels, the greater the resolution [29,30,31].

2. *Contrast* is the difference in signal intensity between a sample and an image’s average signal intensity. As with resolution, the concept of contrast has implications for the ability to discern between different structures [32].

3. In general, *dynamic range* refers to when a variable has two values that have a significant difference, which relates to the difference between the most and least extreme signals (most likely from a sample) when imaging (from the background) [33].

4. The *signal-to-noise ratio* (SNR) is a general metric for determining image quality. It is described as the relative strength of an aimed signal from a sample compared with the undesired background signal from noise. SNR must be high enough to distinguish a sample from the background, which relates to the concepts of contrast, resolution, and dynamic range [31,34].

#### Appendix A.1.2. Graphical Representation of Digital Image

The representation of an image can take several forms, which can vary in regards to their color space (including aspects such as hue, saturation, or value) and even in graphical schemes. This representation conveys information, such as color, coded information, temperature mapping, and how an image is digitally preserved.

##### Histogram

A histogram is defined as a graphical representation of the frequency of an event’s occurrence. Image processing presents a relative frequency of occurrence of various gray levels (which can be shown with a bar chart representation). The histograms of images also provide a global description of their appearances [35]. The global description displays the number of pixels in each gray level but not their location as spatial coordinates (local information). Finally, different images can be generated from the same histogram, as pixel location is not maintained in a histogram; therefore, a histogram is not a unique representation of images. It only counts the pixels of each gray level.

In HE, the perfect image is one where each gray level has the same number of pixels. The main objective of HE is to have equal pixels at all gray levels, not just to distribute the dynamic range. It is impossible to obtain an exactly equalized image from a digital image. In HE, a decision is taken based on two parameters: the density (or the number of pixels) of gray levels and the transfer function. HE reallocates the cumulative histogram used as a transfer function to distribute the pixel intensity levels evenly. The purpose of HE is to produce an output image with a histogram that has a flattened result. The objective of histogram matching is to take an input image and generate an output image based on the shape of a specified (or reference) histogram.

##### CDF

A required function derived from a histogram is called a cumulative histogram. Figure A1 shows the concept of a cumulative histogram. There are two intervals with the same widths but different slopes; one interval has a gentle slope, and the other has a steep slope. The number of pixels is accumulated for each intensity value. The density of a steep slope is higher than that of a gentle slope. The projected interval is based on the slope of the cumulative histogram.

The numerical data (i.e., the frequency distribution of pixels) that are not categorical have distributions. In general, when data are not categorized, the frequency of each entry is ineffective as a summary, since the bulk of entries are unique. Therefore, another useful technique to define a distribution for numeric data is providing a proportion of data below the random variable, which is picked from the histogram of the image. CDF [27] is the term used to describe this function [36]. Mathematically, a CDF is defined as follows:

*F*(*a*) = *Pr* (*x* ≤ *a*) (A1)

where *Pr* is the probability of distribution of *x* (given function). Figure A2 provides a sample image of a *CDF*, demonstrating that a significant number of *F*(*x*) values from a given set of numbers (*a*) are less than or equal to *x*. In addition, when the histogram is perfectly equalized (i.e., when each intensity roughly corresponds to the same number of pixels), the CDF will look like a straight 45° line.

#### Appendix A.1.3. Factors That Affect Image Quality

The assessment of “image quality” is difficult to describe, because it frequently depends on context and application specifics. The primary goal of image quality assessment is developing computational models for assessing perceptual image quality [37]. As seen by human observers, image quality and threshold values are measurable and constant properties, even when comparing images with varying content and types of degradation. Therefore, changes in the application vary in the selected parameters.

#### Appendix A.1.4. Image ED Methods for Image Segmentation

##### Image Segmentation

Using image ED methods, object boundaries can be established on a per-image basis. These methods can be helpful for examining individual pixels and their nearby segments to determine portions of an image that have strong contrast. The method of identifying edges in image processing is known as ED [20]. Image segmentation is a necessary step in image analysis. The segmentation process separates an image into its components or objects that have the same texture or color. The image segmentation process produces a set of regions that span the entire image’s set of contours, which were extracted from the image. Each pixel in a region has certain qualities, such as color, intensity, or texture. The segmentation process initially establishes the boundaries between regions based on discontinuities in intensity levels, followed by thresholds based on pixel property distributions, such as intensity values, and finally directly locates the regions.

##### ED Techniques

ED is an essential preprocessing method for image segmentation. ED techniques convert original images into edge images. ED in image processing, especially in computer vision, is concerned with the localization of significant changes in a gray-level image and detecting physical and geometrical features of objects in a scene [38]. It is a basic technique that recognizes and outlines an object, the borders between objects, and the backdrop in an image. There are many ED techniques; the commonly known ones are Sobel ED (SED), Robert’s ED (RED), Prewitt ED (PED), the Laplacian of Gaussian ED (LOG-ED), and Canny ED (CED). Below, each technique is briefly discussed.

a. SED

The Sobel operator is a fundamental first-order ED operator. As shown in Figure A3, the Sobel operator uses two 3 × 3 convolution masks; the second mask is a 90° rotation of the first mask. Each mask responds to the edges as much as possible, both horizontally and vertically [23]. To obtain the gradients in the right directions, the masks are moved horizontally and vertically. Equations (A2) and (A3) show the magnitude and direction of the gradient, respectively.

*G_mag_* = (*G_x_*^2^ + *G_y_*^2)^)^1/2^
(A2)

*Gdir* = tan^−1^ (*G_y_*/*G_x_*) (A3)

b. RED

RED is a straightforward and efficient approach to quantifying an image’s spatial gradient. The pixel value at a location in the produced image represents the estimated absolute magnitude value of the inputted image’s spatial gradient at that location [39]. It takes a grayscale image as input and generates edges involving the image. However, its functionality is limited by the fact that it is not symmetric and cannot be generalized to identify edges that are multiples of 45°. Figure A4 depicts a pair of 2 × 2 convolution masks of RED. The gradient magnitude is given by Equation (A4), and its corresponding angle of orientation to the spatial gradient is given by Equation (A5).

Δ*f* = *mag*(Δ*f*) = (*G*_*x*_^2^ + *G*_*y*_^2^^)^)^1/2^ (A4)

(A5)
$$\theta =\tan^{-1}\;(G_{y}/G_{x})-\frac{3\Pi }{4}$$

c. PED

The PED algorithm is appropriate for detecting the magnitude and orientation of edges. The PED has the same parameters as the SED, but it is much easier to implement; however, the result is a little noisier, because it differentiates in one direction and averages in another direction [39]. Figure A5 depicts a sample of PED with 3 × 3 convolution masks. Equations (A6) and (A7) show how the Prewitt operator is measured.

The pixel matrix is shown below:

$$\left[\begin{array}{ccc} a_{0} & a_{1} & a_{2}\\ a_{7} & \left[i-j\right] & a_{3}\\ a_{6} & a_{5} & a_{4} \end{array}\right]$$

*G_x_* = (*a*_2_ + *ca*_3_+ *a*_4_) − (*a*_0_ + 2*a*_7_+ *a*_6_)
(A6)

*G_y_* = (*a*_6_ + *ca*_5_+ *a*_4_) − (*a*_0_ + 2*a*_1_+ *a*_2_)
(A7)

d. LOG-ED

A well-known ED approach is the LOG-ED. The LOG-ED first smoothens an image and then calculates the Laplacian. The process results in a double-edged image. It finds edges and then locates the zero-crossing between the double edges [24]. Figure 8 shows 3 × 3 convolution masks of the LOG-ED. Equation (A8) shows the second-order derivative of the LOG-ED.

(A8)
$$\nabla^{2}f=\frac{\delta^{2}f}{\delta x^{2}}+\frac{\delta^{2}f}{\delta y^{2}}$$

e. CED

This is the most common, powerful, and widely used ED approach. Before extracting edges, it isolates the noise from the image. CED is superior to other EDs, and it yields good results [39]. The Canny operator has complete control over various edge image details and can effectively suppress noise. As shown in Figure A7, CED uses a pair of 3 × 3 convolution masks. In addition, Equations (A9) and (A10) provide the local gradient value of CED and its corresponding direction angle, respectively.

*g*(*x*, *y*) = (*G_x_*^2^ + *G_y_*^2)^)^1/2^ (A9)

*α*(*x*, *y*) = tan^−1^(*G_y_*/*G_x_*) (A10)

Most ED techniques use the concept of convolution with a series of directional derivative masks. Gradient-based ED entails calculating the gradient’s (derivative’s) magnitude and comparing it with a fixed threshold to determine the edge points. The numerical gradient is approximated independently in the horizontal *G_x_* and vertical *G_y_* directions.

An edge is a contour in an image along which the brightness of the image quickly shifts. Discontinuities may cause an intensity edge in the normal surface, depth, reflectance, or lighting. To locate edges within an image, ED operators analyze the gray level of each pixel and its neighboring pixels to determine which pixels correspond to regions of high contrast in gray-level intensity. The fundamental ED operator is calculated by constructing a matrix centered at a pixel selected as the matrix area’s center. If the value of this matrix area is more significant than a predefined threshold, the center pixel is considered to be an edge. Typically, the slope and direction of the edge, often referred to as the magnitude and orientation of the gradient vector, respectively, are employed to define the contrast regions. Gradient-based ED determines edge sites by estimating the gradient magnitude in the first derivative and comparing it with a preset threshold. At edge pixels, a rapid rate of change in intensity in the direction specified by the gradient vector’s angle is noticed. The gradient’s magnitude shows the edge’s strength.
